# Impacts of working environment and benefits packages on the health professionals’ job satisfaction in selected public health facilities in eastern Ethiopia: using principal component analysis

**DOI:** 10.1186/s12913-019-4317-5

**Published:** 2019-07-16

**Authors:** Hailu Merga, Tilahun Fufa

**Affiliations:** 1 Department of Epidemiology, Institute of Health, Jimma University, Jimma, Ethiopia; 2 Department of Health Service, Management and Policy, Institute of Health, Jimma University, Jimma, Ethiopia

**Keywords:** Health professionals, Level of job satisfaction, benefitsPackages, Working environment, Ethiopia

## Abstract

**Background:**

World Health Organization (WHO) predicted that there will be a shortfall of skilled healthcare by 2035 with the greatest shortfall in Africa and Southeast Asia due to satisfaction with payment and incentives. Low job satisfaction of health workers can result in increased staff turnover and absenteeism, which affects the efficiency of health services. Ethiopia has been affected by a shortage of health professionals due to a brain drain of health professionals. Our study, therefore, aimed at assessing the impact of the working environment and benefits packages on the level of satisfaction among health professionals working in selected public Health facilities in Eastern Ethiopia.

**Methods:**

Institutional based Cross-sectional study design was conducted among 422 selected health professionals in Bale Zone Public Health Facilities. After selecting 2 hospitals and 32 health centers by lottery method, proportional allocation of the sample was done for selected Hospitals and Health Centers. Then, to select individual health professional from each health center and hospital, a systematic sampling method was employed using the worker’s registration log book. Then, data were collected, cleaned and entered into EpiData software version 3.1 and then exported to IBM SPSS version 21 for analysis. Both descriptive and inferential statistics were done. The principal component analysis was employed for all Likert scale instruments to extract factor(s) representing each of the scales and have factor scores, which facilitate treatment of the variables as continuous during further analysis. Using this regression factor score, multiple linear regression analysis was performed and the effect of independent variables on the regression factor score of the outcome variable was quantified. A significance level of less than 0.05 was used in all cases to judge statistical significance.

**Result:**

This study showed that the prevalence of job satisfaction of health professionals was 38.5% (95%CI: 33.82–43.2%). Age of health professionals ((β = 0.252, (95% CI: 0.067, 0.437))), type of health facility (β = − 0.280, (95% CI; − 0.519, − 0.041), service year (β = 0.487, (95%CI: 0.025, 0.998)), supply they need to do their job (β = 0.10, (95% CI: 0.009 to 0.19)), perception of health professional on allowances (β = − 0.216, (95% CI: − 0.306, − 0.125)) and perception of health professionals on employment benefits (β = 0.225, (95% CI: 0.135 to 0.315)) were statistically significant that affect job satisfaction factor score.

**Conclusion:**

level of job satisfaction of health professionals was found to be low. Level of job satisfaction was influenced by the age of the health professionals, type of health facility in which they were working, years of service they had in the health sectors, their working environment, professional allowance and benefits like financial rewards and benefits of being employed. Hence, policy makers and health managers need to pay special attention to increase the satisfaction of the health workforce at all levels in the health system. Moreover, special emphasis should be given for the benefits packages of health workers at different levels.

**Electronic supplementary material:**

The online version of this article (10.1186/s12913-019-4317-5) contains supplementary material, which is available to authorized users.

## Background

World Health Organization (WHO) predicted that, as the world population increases, there will be a shortfall of 12.9 million skilled healthcare by 2035 with the greatest shortfall in Africa and Southeast Asia. The report indicated that about 40% of health professionals will leave health employment in the next decade due to low pay and too few incentives and it has serious implication for the world population. Similarly, internal and international migration of health workers is an exacerbating regional imbalance of health professionals [1]. As a result of advancement in medical technology and the demand for more sophisticated care, the health system requires a more skilled workforce. WHO report indicates that health workers are the backbone of all health care systems to provide health care and manage health programs and respond to health emergencies [2].

Job satisfaction, a pleasurable or positive emotional state resulting from the appraisal of job experiences, among healthcare workers is increasingly being recognized as a measure that should be included in quality improvement programs in health care delivery [2]. It largely determines the productivity and efficiency of Health institutions. Human resource in the Health system is the most valuable asset and it works as an engine to provide a sustainable service delivery [3, 4].

Evidence showed that the human resources crisis in low-income countries has received global attention, particularly in Sub-Saharan Africa. In some countries less than 50% of the required staff are available to serve the rural populations; while at times care is provided by non-qualified staff, which affects the health status of the community, particularly the poor [2]*.* Moreover, besides the health status of the community, lower job satisfaction among those skilled health workers can result in increased staff turnover and absenteeism, which affects the efficiency of health services. According to WHO identification currently many developing countries, including Ethiopia, the threshold of health workforce density is below the expected [2, 5, 6].

In many countries, health institutions pay close attention to the subjective well-being of their Health workers and its impact on their jobs. The subject of job satisfaction is particularly relevant and of interest to public health practitioners due to the fact that organizational and employees’ health and well-being rest a great deal on job satisfaction. The magnitude of job satisfaction of health workers around the globe varies. Evidence from Norway showed that job satisfaction among doctors was high and increasing, whereas finding from German showed that the satisfaction of doctors increased, but that of Nurses decreased [4, 7–9]. Similarly, finding from Saudi Arabia revealed there was a low level of job satisfaction [10]. Evidence from Ethiopia indicates that the country has been affected by a shortage of health professionals due to a brain drain of health professionals [11].

Working environment and benefits packages are the most important factors for job satisfaction that leads to the turnover in addition to the reduction in quality of health care services. Work environment generally could be described as the place, conditions and surrounding influences in which health workers carry out their activity. Poor working environment leads to greater impact on quality, effectiveness and work efficiency and at the same time on healthcare costs [9, 12, 13]. Different findings showed that Job satisfaction of health professionals is said to be linked with their years of experience, age, professional category, work environment, incentive, job recognition, and promotion opportunities, workloads and relation of employees with their supervisors [4, 14, 15]. Cross-sectional Studies from Western Ethiopia [16] and Addis Ababa [17] showed that compensation and benefits packages were the factors that affect the health professionals` job satisfaction. Similarly, findings from the Harari region [18] and Amhara region of Ethiopia [14] as well as other parts of African countries like South Africa, Malawi and Tanzania [19] revealed that service year, type of health facility in which they work, age of health workers, benefit packages and resource availability were the factors that affect the health workers job satisfaction.

Job satisfaction and motivation of health workers were taken as the challenges to achieve Sustainable Development Goals [20–22] and factors affecting job satisfaction among health professional varies from time to time and place to place.

So far, no study has been done on Job satisfaction among health professionals in Bale Zone of Eastern Ethiopia after the health system reforms started which aimed at provision of safe, effective, convenient and affordable service. Since the reforms, people have been using the health services at a higher rate, leading to an increased burden for health professionals at all levels and that might have decreased their job satisfaction. Besides, the geographical differences among regions of Ethiopia may have an impact on the mobility or internal migration of health workers. Besides, in Ethiopia, the benefits packages paid for all professionals are almost the same irrespective of the geographical difference. This may lead to low satisfaction with their job and low commitment which ultimately reduces the quality of service. Our study, therefore, aimed at assessing the impact of the working environment and benefits packages on the level of satisfaction among health professionals working in selected public Health facilities in Eastern Ethiopia. The finding of this study is important in the identification of the impact of working environment and benefits packages on the level of satisfaction of health professionals to provide quality and efficient health services.

## Methods

### Study design and setting

Institutional based Cross-sectional study design was conducted in Bale Zone, Oromia regional state, Public Health Facilities from 22 April 2017 to 10 May 2017 which is located at the 420 km to the South East of Addis Ababa, the capital. The zone has a total population of 1, 822, 898 peoples in which about 49.5% were females. It was divided into two town administrations and 18 districts. There were about 800 health professionals and 84 public health facilities including 4 governmental hospitals.

### Study participants

All randomly selected health professionals holding a diploma and above qualification and who were working in public hospitals or health centers and those who had 6 months and above work experiences in the study institutions were our source population. Whereas all health workers, working as full time in selected hospital and health centers in Bale zone having a 6-month and greater work experience participated in the study. All health workers who had a work experience of less than 6 months and those who were on sick leave, annual leave and maternity leave at the time of the study were excluded.

### Sample size determination and sampling technique

The sample size was determined using single population proportion formula by considering 44.2% proportion of health workers` job satisfaction from the study conducted in Harari region [18], 95% confidence level and 0.05 margin of error. Then, the sample size became 378. Since the sample size was greater than 5% of the total population and the source population was 10,000, finite population correction formula was used and the sample size became 281. Multistage sampling method was used and the probability of all health professionals in the zone to be included in the sample was not equal. Therefore, to overcome this problem we have increased our sample size by taking design effect 1.5 and the final sample used to collect data was 422. Two hospitals and 32 health centers were selected randomly by lottery methods. Then, the proportional allocation of the sample was done in selected Hospitals and Health Centers. To select an individual health professional from each health center and hospital, a systematic sampling method was employed using the worker’s registration log book. The first health worker was selected by lottery method. The pretested data collection instrument was adopted from the JHPEIGO national strengthening human resources for health and related relevant literature (Additional file 1). The questionnaire mainly consisted of close-ended questions addressing socio-demographic characteristics of respondents, Job satisfaction, working and living conditions, and Perceived of compensation and benefits.

### Measurements

#### Job satisfaction

Job satisfaction was assessed using 22 items on a five-point Likert scale ranging from very dissatisfied [1] to very satisfied [5]. These 22 items were based on the following questions: satisfaction with salary package, salary fairness compared to other staffs, opportunity for promotion, fair benefit, job match with skill, presence of clear job description, getting recognition, fair application of personal policy by the supervisor, work plan developed with supervisor, annual performance appraisal, organization value, supervisor support on work, organization encourage family, training opportunity, coaching and mentoring, measures to protect from HIV/AIDS and injury, perceive as part of local community, community value for work, competency of managers of health facility, good relationship with co-worker and intention to continue work in the health facility for at least for 2 years. The reliability coefficient (Cronbach’s Alpha) of the job satisfaction scale was 0.926 indicating the scale was internally highly consistent. To extract the underlying factors (components) of the job satisfaction scale, Principal Component Analysis (PCA) was conducted and one meaningful factor with an eigenvalue greater one was extracted. Principal component analysis (PCA) is a technique used to emphasize variation and bring out strong patterns in a dataset and often used to make data easy to explore and visualize. During the analysis, the scale was reduced to one item (Job satisfaction score) with an eigenvalue greater than one and 0.906 KMO (P = < 0.001). The extracted item explained 72.45% of the overall variance and was used during further analysis as a continuous variable.The prevalence of job satisfaction in this study was calculated using the percent mean formula: actual value minus potential minimum divided by Potential maximum minus potential minimum [23–26].

#### Working and living conditions

It was assessed using 15 items on a five-point Likert scale ranging from strongly disagree [1] to strongly agree [5]. This scale was found to have high internal consistency (Cronbach’s alpha =0.746). These 15 items were based on the following questions: reasonable workload, supply needs to do job, working equipment needed, access to drug and medication, clean workspace, time to eat lunch almost every time, safe and clean water at home, safe and clean water workplace, access to electricity at workplace, access to electricity at home, good internet connection at workplace, access to good schooling for children, safe and efficient transportation, worry about losing job and good shopping and entertainment. The reliability coefficient (Cronbach’s Alpha) of the working and living condition scale was 0.795 indicating the scale was internally consistent. To extract the underlying factors (components) of the working conditions, Principal Component Analysis (PCA) was conducted and one meaningful factor with eigenvalue greater one was extracted. During the analysis, the scale was reduced to one item (working condition) with an eigenvalue greater than one and 0.668 KMO (P = < 0.001). The extracted item explained 77.08% of the overall variance and was used during further analysis as a continuous variable.

#### Perceived of compensation and benefits

It was assessed using nine items on a five-point Likert scale ranging from not important [1] to extremely important [5]. This scale was found to have consistency (Cronbach’s alpha = 0.895). These 15 items were based on the following questions: salary, terminal benefit, housing allowance, assistance with transportation, risk allowance, duty allowance, health care for family, professional risk allowance and food allowance. To extract the underlying factors (components) of the working conditions, Principal Component Analysis (PCA) was conducted and one meaningful factor with eigenvalue greater one was extracted. During the analysis, the scale was reduced/renamed to two items (professional allowance and employee benefits) with an eigenvalue greater than one and 0.856 KMO (P = < 0.001). The extracted items (professional allowance and employee benefits) explained 84.27 and 77.14% of the overall variance respectively and were used during further analysis as a continuous variable. Finally, the questionnaire prepared in English was translated into regional language Afan Oromo and retranslated back into English to ensure its consistency. Six trained diploma graduated health professionals native Afan Oromo speakers facilitated the data collection process. Data collectors were trained for one day to be familiar with the data collection tool. Editing and sorting of the self-administered questionnaires were done to determine the completeness and consistency of data every day during the data collection.

#### Analysis

STROBE checklist was used to analyze and report data [27]. Data were cleaned and entered into EpiDatasoftware version 3.1 and then exported to IBM SPSS version 21 for analysis. Both descriptive and inferential statistics were done. For the socio-demographic characteristics, descriptive statistical analysis was used. Principal Component Analysis was employed for all Likert scale instruments to extract factor(s) representing each of the scales and have factor scores, which facilitate treatment of the variables as continuous during further analysis. Using this regression factor score, the statistical significance of each variable was checked using single linear regression. Those variables with *p*-value less than 0.25 were entered into the final model. Then, multiple linear regression analysis was done to identify the determinants of satisfaction. All assumptions of multiple linear regressions were checked. Then, variables with *p*-value less than 0.05 were taken to judge statistical significance. Eigen value of one and above extraction and varimax rotation methods were used during the procesdure of Pricipal Component Analysis procedures. Factors with Cronbach’s alpha value above 0.7 were employed in the subsequent analysis. Factors were renamed in case the scale had above one extracted factors.

## Results

### Sociodemographic characteristics of respondents

Out of 422 populations included in the sample, 415 participated in the study yielding a response rate of 98.3%. One hundred eighty-five (44.6%) of the respondents were under the age of 25 years with the mean(+SD) age of 26. 98 (+ 5.34) years. Two hundred fifty (60.2%) respondents were males. The dominant ethnic group was Oromo (84.1%) followed by Amhara. About two-thirds (62.2%) and about one fourth (24.8%) of the respondents were followers of Protestant and Orthodox religions respectively. Two hundred sixty-five (64%) of study participants were married while about one third (32.3%) were single. Among the respondents, more than half (58.8%) were diploma holders, while 39% of respondents were degree holders at their educational level. Two hundred fifty-one (60.5%) of the study participants were nurses or midwives in their profession. Concerning year of service in the health sector, more than half (59.5%) of respondents had 1–5 years while about one fourth (25.5%) of them had 6–10 years. Two hundred fifty-three (61%) of the respondents were living in the rental house while only 16.4% of respondents were living in their own house. This study revealed that the majority (81.2%) of respondents were working in the health centers (Table 1).

**Table 1:** Socio-demographic and socioeconomic characteristics of health workers in Bale Zone, Eastern Ethiopia, 2017

Variable	Category	Frequency	Percentage
Age	< 25	185	44.6
	25–29	132	31.8
	30–34	61	14.7
	35–39	25	6.0
	Above 39	12	2.9
Sex	Male	250	60.2
	Female	165	39.8
Ethnicity	Oromo	349	84.1
	Gurage	7	1.7
	Tigre	11	2.7
	Amhara	44	10.6
	Others^a^	4	1.0
Religion	Orthodox	103	24.8
	Catholic	19	4.6
	Muslim	29	7.0
	Protestant	258	62.2
	Others^b^	6	1.4
Marital status	Single	134	32.3
	Married	265	63.9
	Divorced	14	3.4
	Widowed	2	.5
Educational level	Diploma	244	58.8
	Degree	161	38.8
	Masters and above	10	2.4
Professional category	Physicians	6	1.4
	Pharmacy and druggists	54	13.0
	Nurses or midwives	251	60.5
	Health officers	42	10.1
	Laboratory Technician and Technologists	39	9.4
	Others^c^	23	5.5
Year of service in Health sector	Below 1 year	48	11.6
	1–5 years	247	59.5
	6–10 years	106	25.5
	Above 10 years	14	4.4
Monthly salary	< 3000	126	30.4
	3000–5000	222	53.5
	> 5000	67	16.1
Status of residential house	Own	68	16.4
	Rent from public	26	6.3
	Rent from private	253	61.0
	provided by health facility	43	10.4
	Live with parents	25	6.0
Type of working Health facility	Health center	337	81.2
	Hospital	78	18.8

^a^ Hadiya and Sidama, ^b^Wakefata, ^c^ Environmental health and postgraduate studies

### Prevalence of health professional job satisfaction

The prevalence of job satisfaction in this study was calculated using the percent mean formula i.e. the actual value minus potential minimum divided by Potential maximum minus potential minimum. First, we computed the score of each variable for each respondent. Then, the mean scores of each variable for each respondent was calculated. Then, the mean of means was performed. Finally, the percent mean was calculated by using the percent mean formula mentioned above. Hence, the prevalence of job satisfaction of health professionals found that 38.5% (95% CI: 33.82–43.2%).

### Predictors of the level of health professionals satisfaction

All predictors of job satisfaction with *p*-value < 0.25 were entered into a multiple linear regression model and the final predictors of the job satisfaction score were identified. The model explains about 62.6% % (p = < 0.001) of the variance in the job satisfaction. Accordingly, the type of facility (*p* = 0.022), service year in the health sector (*p* = 0.006), age(*p* = 0.008), availability of supply needed for their work (*p* = 0.031), perceived professional allowances (p = < 0.001) and perceived benefits of being employed (p = < 0.001)of that institution score were appeared to be statistically significant that affect job satisfaction factor score.

Health professionals who were working in the hospital had 0.28 unit decreased in satisfaction score when compared to those who were working in health centers (β = − 0.280, (95% CI; − 0.519, − 0.041).This study revealed that health professionals who had 10 years and above service years had 0.487 unit more satisfaction than those with 5 years and below (β = 0.487, (95%CI: 0.025, 0.998)). Health professionals whose age were less than 25 years old had 0.252 unit greater satisfaction score when compared to those whose age were between 25 to 34 years (β = 0.252, (95% CI: 0.067 to 0.437)). Respondents who got the supply they need to do their job had an average increase of 0.1unit in their job satisfaction score (β = 0.10, (95% CI: 0.009 to 0.19)). As the perception of health professional allowances increased by one unit, job satisfaction decreased by 0.216 (β = − 0.216, (95% CI: − 0.306, − 0.125)). As the perception of health professionals on employment benefits increased by one unit, their job satisfaction increased by 0.225 (β = 0.225, (95% CI: 0.135 to 0.315)) (Table 2).

**Table 2:** Predictors of level of Health Professionals satisfaction in Bale Zone, Eastern Ethiopia, 2017

Variable	Category	Frequency, N (percentage, %)	Unstandardized coefficients	Standardized coefficients	*p*- value	Confidence interval
			B	Beta		Lower, Upper
Type of facility	Health center	337(81.2)				
	Hospital	78(18.8)	−0.280	−0.109	0.022	−0.519, −0.041
Year of service provision	5 and below years	295 (71.1)				
	6–10 years	106 (25.5)	−0.950	−0.042	0.398	−0.316, 0.126
	Above 10 years	14(4.4)	0.487	0.088	0.006	0.025, 0.998
Age	Less than 25 years		0.252	0.125	0.008	0.067, 0.437
	25–34 years					
	Above 34 years		−0.222	−0.063	0.196	−0.559, 0.115
Availability of supply needed for their work			0.100	.0.100	0.031	0.009, 0.190
Professional allowance			−0.216	− 0.216	0.000	− 0.306, − 0.125
Benefits of being employed			0.225	0.225	0.000	0.135, 0.315

## Discussion

This study clearly revealed that the magnitude of job satisfaction in Eastern Ethiopia was 38.5% (95% CI: 33.82–43.2%). This result is higher than the study done in the Amhara region of Ethiopia [14] and Pakistan [28] which were 31.7 and 18% respectively. The difference might be due to the study setting and working environment as well as working condition. But, the result is almost consistent with the study done in Western Ethiopia [16] and southwestern Ethiopia [21] which were 41.46 and 41.4% respectively as well as the finding from China [29]. However, the finding of our study is lower than the study from Addis Ababa, 52.9% [17] and Harari region, 44.2%, Ethiopia [18]. Similarly, a study conducted in four regions of Ethiopia [30] revealed that 79.5% of health workers were satisfied with the job they were performing. This might be due to the difference in the study settings, our study included both public Health facilities found in urban and rural areas; while, these studies included only health facilities found in large towns or cities. Besides, the difference might be due to some workers whose place of residence is nearby health institutions are more satisfied as they may have no plan to move somewhere to seek jobs. Moreover, workers in a big city might be more interested in job satisfaction than those in a small city. Furthermore, the difference might be occurred due to the natural difference peoples have for satisfaction; which may increase or decrease the magnitude of satisfaction. Again, our finding is lower than the reports from Malawi (71%), South Africa (52.1%), Tanzania (82%) and Pakistan (79.88%) [19, 31]. The difference might be due to the difference in sociodemographic, socioeconomic and difference in study settings.

Our study revealed that, the age of the health professionals, type of health facility in which they were working, years of service they had in the health sector, work environment like resource availability for work, professional allowance and benefits like financial reward and Employment benefits like benefits for being employment of the health institution were statistically significant.

Health professionals whose age were less than 25 years old had 0.252unit greater satisfaction score when compared to those whose age were between 25 to 34 years. This study is in line with studies conducted in the Harari region of Ethiopia [18], Tanzania [19, 32], Pakistan [31] and Serbia [33].

This study revealed that health professionals who had 10 years and above service years in the health sector had 0.487 unit more satisfaction than those with 5 years and below. This finding is supported by findings from the Amhara region of Ethiopia [14], Serbia [33], Pakistan [31] and China [29]. These findings clearly showed that the satisfaction level of health workers was increased with their service years in the health sector. This might be due to the reason that as the service year in the health sector increases, health workers become more stable, which may result in more job satisfaction.

Health professionals who were working in the hospital had 0.28 units decreased in satisfaction score when compared to those who were working in health centers. This result is in agreement with the finding from the Harari region of Ethiopia, which showed more satisfaction among health workers working in Health centers [18]. The study also agreed with the findings from Tanzania, Malawi and South Africa [19]. The underlined similarity might be due to the fact that workers working in the hospital may fear for high workload and the probability of infection due to the nature of hospital service provision style.

In our study, respondents who got the supply they need to do their job had an average increase of 0.1 units in their job satisfaction score. The finding is similar to the finding of the researches from Ethiopia [30] and Tanzania [32]. This might be due to the unavailability of resources for service provision directly influences the satisfaction of health professionals. Moreover, the availability of resources (job aids) is critical for the improvement of the health professionals work process and in supporting communication with service users.

This study showed that the perception of health professionals towards allowances and employment benefits had a direct relationship with job satisfaction. These were not statistically significant in the other previous studies [16]. The difference might be due to the fact that there could be a difference in the health institutions management system and behavior as well as the difference in the amount of benefits and packages in those health institutions.

### Limitations

The study used a standardized tool to measure satisfaction level and used Principal Component Analysis for the variable reduction. However, this study was not without limitation: although the standardized tool was used for measurement and participants were assured of confidentiality, there was a possibility of under or over-reporting their level of satisfaction. Notwithstanding this limitation, we believe that our study has very important findings for strengthening the Human workforce for the current health system in the study area and areas with similar setups.

## Conclusion

In conclusion, our finding revealed that the level of job satisfaction of health professional was low. It showed that the working environment and benefits packages had an impact on the health professionals` job satisfaction. Moreover, the level of job satisfaction was influenced by the age of the health professionals, type of health facility in which they were working, and years of service they had in the health sectors. Hence, policy makers and health managers need to pay special attention to increase the satisfaction of the health workforce at all levels in the health system. Moreover, special emphasis should be given for the benefits packages of health workers at different levels.

## Additional file

Additional file 1: Quesionnaire to assess impacts of Working Environment and Benefit packages on the Health professionals’ Job satisfaction. (DOCX 33 kb)

## Data Availability

The datasets used and/or analysed during the current study are available from the corresponding author on reasonable request.

## Abbreviations

CI: Confidence Interval
JHPEIGO: Johns Hopkins Program for International Education in Gynecology and Obstetrics
*KMO*: Kaiser-Meyer-Olkin
OR: Odds Ratio
PCA: Principal Component Analaysis
SPSS: Statistical Package for Social Sciences
WHO: World Health Organization

## References

[1] Global Health Workforce Alliance and World Health Organization (2013). A Universal Truth: No Health Without a Workforce Executive Summary.

[2] Dieleman M, Harnmeijer JW. Improving health worker performance: in search of promising practices [Internet]. WHO; 2006. p. 1–71. Available from: https://www.who.int/hrh/resources/improving_hw_performance.pdf. Accessed 21 Dec 2018.

[3] Saari LM, Judge TA (2004). Employee attitudes and job satisfaction. Hum Resour Manag.

[4] Timalsina R, Rai L, Gautam S, Panta P. Factors Associated With Organizational Commitment among Nurses. J Stud Manag Plan. 2015;1(7) Available from: http://internationaljournalofresearch.org/index.php/JSMaP. Accessed 26 Nov 2018.

[5] Nikic D, Arandjelovic M, Nikolic M, Stankovic A. Job satisfaction in health care workers. Acta Medica Medianae. 2008;47:9–12. [Google Scholar].

[6] Ramasodi JMB. Factors Influencing Job Satisfaction Among Healthcare Professionals at South Rand Hospital. Johannesburg: University of Limpopo (Medunsa Campus), 2010;144. Available from: https://books.google.com.et/books?id=5cHVYgEACAAJ. Accessed 25 Nov 2018.

[7] Aasland Olaf G., Rosta Judith, Nylenna Magne (2010). Healthcare reforms and job satisfaction among doctors in Norway. Scandinavian Journal of Public Health.

[8] Alameddine Mohamad, Bauer Jan Michael, Richter Martin, Sousa-Poza Alfonso (2015). Trends in job satisfaction among German nurses from 1990 to 2012. Journal of Health Services Research & Policy.

[9] Bahalkani HA, Kumar R, Lakho AR, Mahar B, Mazhar SB, Majeed A. Job satisfaction in nurses working in tertiary level health care settings of Islamabad, Pakistan. J Ayub Med Coll Abbottabad. 2011;23(3):130–3. [PubMed] [Google Scholar]. 23272454

[10] Hamasha AA, Alturki A, El-metwally A (2019). Predictors and level of job satisfaction among the dental workforce in National Guard Health Affairs Abstract Objective : materials and methods : results : conclusions. J Int Soc Prev Community Dent.

[11] Girma S, Yohannes A, Kitaw Y, Ye-Ebiyo Y, Seyoum A, Desta H, et al. Human Resource Development for Health in Ethiopia: Challenges of Achieving the Millennium development Goals. Ethiop J Heal Dev. 2008;21(3) Available from: http://www.ajol.info/index.php/ejhd/article/view/10052. Accessed 19 Dec 2018.

[12] Wiskow C, Albreht T, De Pietro C (2010). Health systems and policy analysis: how to create an attractive and supportive working environment for health professionals.

[13] Chaulagain N, Khadka DK. Factors Influencing Job Satisfaction Among Healthcare Professionals At Tilganga Eye Centre, Kathmandu, Nepal. 2012;1(11):32-36.

[14] Temesgen K, Aycheh MW, Leshargie CT (2018). Job satisfaction and associated factors among health professionals working at Western Amhara region, Ethiopia. Health Qual Life Outcomes.

[15] Kumar R, Ahmed J, Shaikh BT, Hafeez R, Hafeez A, Kumar R, Ahmed J, Shaikh B, Hafeez R, Hafeez A (2013). Job satisfaction among public health professionals working in public sector: a cross sectional study from Pakistan. Hum Resour Health.

[16] Deriba BK, Sinke SO, Ereso BM, Badacho AS (2017). Health professionals ’ job satisfaction and associated factors at public health centers in West Ethiopia. Hum Resour Health.

[17] Bekru ET, Cherie A, Anjulo AA (2017). Job satisfaction and determinant factors among midwives working at health facilities in Addis Ababa city, Ethiopia. PLoS One.

[18] Geleto A, Baraki N, Atomsa GE, Dessie Y (2015). Job satisfaction and associated factors among health care providers at public health institutions in Harari region, eastern Ethiopia : a cross - sectional study. BMC Res Notes.

[19] Blaauw Duane, Ditlopo Prudence, Maseko Fresier, Chirwa Maureen, Mwisongo Aziza, Bidwell Posy, Thomas Steve, Normand Charles (2013). Comparing the job satisfaction and intention to leave of different categories of health workers in Tanzania, Malawi, and South Africa. Global Health Action.

[20] CSA and ICF International (2012). Ethiopia Demographic and Health Survey 2011.

[21] Yami A, Hamza L, Hassen A, Jira C, Sudhakar M (2011). Original article job satisfaction and its determinants among health workers in Jimma University specialized hospital, Southwest Ethiopia. Ethiop J Heal sceinces.

[22] Bergstrom BA, Lunz ME (1998). Measuring job satisfaction: reliability of subscale analysis.

[23] Yang GW, Yuan RX, Huang XF (2011). The essence of governance in health development. Int Arch Med.

[24] Feyissa GT, Abebe L, Girma E, Woldie M (2012). Validation of an HIV-related stigma scale among health care providers in a resource-poor Ethiopian setting. J Multidiscip Healthc.

[25] Feyissa GT, Abebe L, Girma E, Woldie M (2012). Stigma and discrimination against people living with HIV by healthcare providers, Southwest Ethiopia. BMC Public Health.

[26] Tateke T, Woldie M, Ololo S (2012). Determinants of patient satisfaction with outpatient health services at public and private hospitals in Addis Ababa, Ethiopia. Afr J Prim Heal Care Fam Med.

[27] Von EE, Altman DG, Egger M, Pocock SJ, Gøtzsche C, Vandenbroucke JP (2007). Policy and practice the strengthening the reporting of observational studies in epidemiology ( STROBE ) statement : guidelines for reporting observational studies. Bull World Health Organ.

[28] Tasneem Saima, Cagatan Ayse Seyer., Avci Mehmet Zeki., Basustaoglu Ahmet Celal. (2018). Job Satisfaction of Health Service Providers Working in a Public Tertiary Care Hospital of Pakistan. The Open Public Health Journal.

[29] Lu Y, Hu X, Huang X, Zhuang X, Guo P, Feng L (2016). Job satisfaction and associated factors among healthcare staff : a cross-sectional study in Guangdong Province, China. BMJ Open.

[30] Hotchkiss DR, Banteyerga H, Tharaney M. Job satisfaction and motivation among public sector health workers : evidence from Ethiopia. Hum Resour Health. 2015;1–12. Available from: doi: 10.1186/s12960-015-0083-6 10.1186/s12960-015-0083-6 PMC4625466 26510794

[31] Paper O (2013). Job satisfaction of health-care workers at health centers in Vientiane capital and Bolikhamsai Province, Lao PDR. Nagoya J Med Sci.

[32] RNM M, Bhatnagar A, Lefevre A, Chitama D, Urassa DP, Kilewo C (2015). Motivation and satisfaction among community health workers in Morogoro Region, Tanzania : nuanced needs and varied ambitions. Hum Resour Health.

[33] Kuburovic Nina, Dedic Velimir, Djuricic Slavisa, Kuburovic Vladimir (2016). Determinants of job satisfaction of healthcare professionals in public hospitals in Belgrade, Serbia - cross-sectional analysis. Srpski arhiv za celokupno lekarstvo.

