# Supplementary material for: Impacts of working environment and benefits packages on the health professionals’ job satisfaction in selected public health facilities in eastern Ethiopia: using principal component analysis
Source: BMC Health Serv Res. 2019 Jul 16;19:494. doi: 10.1186/s12913-019-4317-5 (PMC6636107; doi:10.1186/s12913-019-4317-5)
Supplement: Supplementary file 1 — Quesionnaire to assess impacts of Working Environment and Benefit packages on the Health professionals’ Job satisfaction. (DOCX 33 kb) [file 12913_2019_4317_MOESM1_ESM.docx]

**English Version Questionnaire**

101. Date of data collection ____________________

102. Name of woreda/town ____________________

**Part II: Socio-demographic characteristics of health workers**Please circle your best choices or fill in the blank spaces

| Q.No | Questions | Possible choice/ Answers |
| --- | --- | --- |
| 201 | Age | 1. <25 4. 45 - 55 2. 25- 35 5. ≥55 3. 35 - 45 |
| 202 | Sex | 1. Male 2. Female |
| 203 | Ethnicity | 1. Oromo 4. Amhara 2. Gurage 5. Other (specify) ________ 3. Tigre |
| 204 | Religion | 1. Orthodox 4. Protestant 2. Catholic 5. Other (specify) ________ 3. Muslim |
| 205 | Marital status | 1. Single 4. Widowed 2. Married 5. Other (specify) _______ 3. Divorced |
| 206 | What is your birth place?  (Determine whether it is urban or rural and circle answer) | 1= Urban  2= Rural |
| 207 | Educational qualification, | - 1. Diploma 3. Masters & above   2. Degree 4. Other (specify) ________ |
| 208 | Professional category. | 1. Physician 5. Health officer 2. Pharmacy & druggists 6. Laboratory 3. Nurses or mid wife 7. Anesthesia 4. Environmental healt 8. Others(specify)______ |
| 209 | Service year in health sector | - - - 1. Below 1year 4. 11–15 years       2. 1–5 years 5. 16–20 years       3. 6–10 years 6. 20 years and above |
| 210 | Service in current health institution | 1. Less than 1year 4. 11–15 years 2. 1–5 years 5. 16–20 years 3. 6–10 years 6. 20 years and above |
| 211 | Average monthly income | 1. <2000 3. 3001 -4000  2. 2001-3000 4. >4001 |
| 212 | Type of facility | 1. Health center 2. Hospital |
| 213 | Family conditions | 1. Living with family 3. No family  2. Living separated from family 4. other ____________ |
| 214 | What is the status of your current residential house? (Circle one) | 1= Own 4. Provide by health facility  2= Rent from public 5. Live with parents  3= Rent from private |
| 215 | Have you specialized or upgraded from your first professional qualification? | 1= Yes 2= No |
| 216 | Do you have a current obligation (compulsory service scheme) to work in the public health system?  Note: Refer to the remaining years and /or months | 1. Yes 2. No |

**Part III: Job Satisfaction**

*Now I want to ask how you feel about your current job. Please tell me whether you agree or disagree with each statement, using a 5 point scale where:*

*5=strongly agree 4=agree 3=neutral 2=disagree 1=strongly disagree*

| # | To what extent do you agree or disagree with the following statements? | 5= Strongly agree | 4= Agree | 3= Neutral | 2= Disagree | 1= Strongly disagree |
| --- | --- | --- | --- | --- | --- | --- |
| 301 | Considering everything, I am satisfied with my job. | 5 | 4 | 3 | 2 | 1 |
| 302 | My salary package is fair | 5 | 4 | 3 | 2 | 1 |
| 303 | My salary is fair compared to other staff with the same level of responsibility. | 5 | 4 | 3 | 2 | 1 |
| 304 | I feel there are sufficient opportunities for promotion with my employer | 5 | 4 | 3 | 2 | 1 |
| 305 | My benefits (such as transportation, duty allowance, housing, etc. ) are fair compared with other staff at my level | 5 | 4 | 3 | 2 | 1 |
| 306 | The job is a good match for my skills and experience | 5 | 4 | 3 | 2 | 1 |
| 307 | My job description is clear and up to date | 5 | 4 | 3 | 2 | 1 |
| 308 | I receive recognition for doing good work. | 5 | 4 | 3 | 2 | 1 |
| 309 | My supervisor applies personnel policies and practices fairly to me | 5 | 4 | 3 | 2 | 1 |
| 310 | I have a current work plan developed with my supervisor | 5 | 4 | 3 | 2 | 1 |
| 311 | My annual performance appraisal is based on my work plan | 5 | 4 | 3 | 2 | 1 |
| 312 | I feel that the organization values my work | 5 | 4 | 3 | 2 | 1 |
| 313 | My supervisor is available when I need support | 5 | 4 | 3 | 2 | 1 |
| 314 | I would encourage my friends and family to seek care here. | 5 | 4 | 3 | 2 | 1 |
| 315 | I have been given the training that I need to succeed in my position | 5 | 4 | 3 | 2 | 1 |
| 316 | I have access to coaching and mentoring when needed | 5 | 4 | 3 | 2 | 1 |
| 317 | The facility takes specific measures to protect me against HIV/AIDS and other occupational hazards | 5 | 4 | 3 | 2 | 1 |
| 318 | I consider myself a part of the local community that I serve as a health worker | 5 | 4 | 3 | 2 | 1 |
| 319 | I feel that the community values my work | 5 | 4 | 3 | 2 | 1 |
| 320 | The head of this health facility is competent and committed | 5 | 4 | 3 | 2 | 1 |
| 321 | I have a good relationship with co-workers | 5 | 4 | 3 | 2 | 1 |
| 322 | Overall, the morale level in my team or work group i s good | 5 | 4 | 3 | 2 | 1 |
| 323 | I intend to continue working here for at least 2 years | 5 | 4 | 3 | 2 | 1 |

**Section IV: Working and Living Conditions**

Now I want to ask you about the working conditions at your current facility. Please tell me whether you agree or disagree with each statement, using a 5-point scale where: 5=strongly agree 4=� agree 3= neutral 2= disagree) 1= strongly disagree

| # | To what extent do you agree or disagree with the following statements? | 5= Strongly agree | 4= Agree | 3= Neutral | 2= Disagree | 1= Strongly disagree | 9=Not applicable |
| --- | --- | --- | --- | --- | --- | --- | --- |
| 401 | My work load is reasonable | 5 | 4 | 3 | 2 | 1 | 9 |
| 402 | I have the supplies I need to do my job well and safely) (Such as gloves, needles, bandages, sutures, disinfectants | 5 | 4 | 3 | 2 | 1 | 9 |
| 403 | I have the working equipment I need to do my job well and efficiently) (Such as ultra sound, x-ray, blood pressure cuffs | 5 | 4 | 3 | 2 | 1 | 9 |
| 404 | This facility has good access to drugs and medications | 5 | 4 | 3 | 2 | 1 | 9 |
| 405 | My work space is clean | 5 | 4 | 3 | 2 | 1 | 9 |
| 406 | I can take time to eat lunch almost everyday | 5 | 4 | 3 | 2 | 1 | 9 |
| 407 | At home, I have access to safe, clean water | 5 | 4 | 3 | 2 | 1 | 9 |
| 408 | At work, I have access to safe, clean water | 5 | 4 | 3 | 2 | 1 | 9 |
| 409 | At home, I have good access to electricity | 5 | 4 | 3 | 2 | 1 | 9 |
| 410 | At work, I have good access to electricity | 5 | 4 | 3 | 2 | 1 | 9 |
| 411 | At work, I have good internet connectivity | 5 | 4 | 3 | 2 | 1 | 9 |
| 412 | I have access to good schooling for my children | 5 | 4 | 3 | 2 | 1 | 9 |
| 413 | I have safe and efficient transportation to work. | 5 | 4 | 3 | 2 | 1 | 9 |
| 414 | I am not worried about losing my job | 5 | 4 | 3 | 2 | 1 | 9 |
| 415 | The community where I live has good shopping and entertainment | 5 | 4 | 3 | 2 | 1 | 9 |

**Section V: Importance of Compensation and Benefits**

Next I want to ask your personal opinion about various compensation and benefits that employers may offer. How important is each of the following factors to you personally in deciding to stay in this job? Use a 5-point scale to answer, where:

5=extremely important, 4=very important, 3=important, 2=somewhat important, 1=not important

| # | How important are the following compensation and benefits factors to you personally in deciding to stay in this job? | 5=  Extremely important | 4=  very  important | 3=  Important | 2=  somewhat important | 1=  Not important |
| --- | --- | --- | --- | --- | --- | --- |
| 501 | Salary | 5 | 4 | 3 | 2 | 1 |
| 502 | Terminal benefits) (such as retirement and pension | 5 | 4 | 3 | 2 | 1 |
| 503 | Receiving a housing allowance or free housing | 5 | 4 | 3 | 2 | 1 |
| 504 | Assistance with transportation | 5 | 4 | 3 | 2 | 1 |
| 505 | Risk allowance | 5 | 4 | 3 | 2 | 1 |
| 506 | Duty allowance | 5 | 4 | 3 | 2 | 1 |
| 507 | Health care for family | 5 | 4 | 3 | 2 | 1 |
| 508 | Professional risk/hazard allowance | 5 | 4 | 3 | 2 | 1 |
| 509 | Food allowance  Note: Food allowance refers to catering service for staff on duty, maternity and OR, etc | 5 | 4 | 3 | 2 | 1 |

**Section VI: Factors that Affect the Job Satisfaction**

Use a 5-point scale to answer, where:

5=extremely important 4=very important 3=important 2=somewhat important 1=not important

| # | If you were not satisfied with current job position, how important would the following factors be? | **5=**  extremely important | **4=**very important | **3=** important | **2=** somewhat important | **1=** not important | 9=Not applicable |
| --- | --- | --- | --- | --- | --- | --- | --- |
| 601 | Low pay | 5 | 4 | 3 | 2 | 1 | 9 |
| 602 | Heavy workload | 5 | 4 | 3 | 2 | 1 | 9 |
| 603 | Long hours of work | 5 | 4 | 3 | 2 | 1 | 9 |
| 604 | Unfair treatment by a supervisor | 5 | 4 | 3 | 2 | 1 | 9 |
| 605 | Poor access to supplies and equipment at work | 5 | 4 | 3 | 2 | 1 | 9 |
| 606 | Limited opportunities for in-service training | 5 | 4 | 3 | 2 | 1 | 9 |
| 607 | Limited opportunities for promotion | 5 | 4 | 3 | 2 | 1 | 9 |
| 608 | Lack of recognition for good work done | 5 | 4 | 3 | 2 | 1 | 9 |
| 609 | Social conflicts in the workplace | 5 | 4 | 3 | 2 | 1 | 9 |
| 610 | Poor supervision and feedback | 5 | 4 | 3 | 2 | 1 | 9 |
| 611 | Concerns about safety at work | 5 | 4 | 3 | 2 | 1 | 9 |
| 612 | Transportation problems | 5 | 4 | 3 | 2 | 1 | 9 |
| 613 | Poor/lack of utilities(water, electricity) at home | 5 | 4 | 3 | 2 | 1 | 9 |
| 614 | Poor/lack of utilities (water, electricity, Internet) at work | 5 | 4 | 3 | 2 | 1 | 9 |
| 615 | Lack of housing facilities | 5 | 4 | 3 | 2 | 1 | 9 |
| 616 | Access to telephones to stay in touch with family and friends | 5 | 4 | 3 | 2 | 1 | 9 |
| 617 | High cost of living | 5 | 4 | 3 | 2 | 1 | 9 |
| 618 | Poor educational facilities for children | 5 | 4 | 3 | 2 | 1 | 9 |
| 619 | Poor access to higher education for yourself | 5 | 4 | 3 | 2 | 1 | 9 |
| 620 | Work is far from home | 5 | 4 | 3 | 2 | 1 | 9 |
| 621 | Poor relationship (unsupportive) with case team leader/CEO/Medical director/ or head of health center | 5 | 4 | 3 | 2 | 1 | 9 |

**Section VII: Additional Questions about turnover**

| Q.N | Questions | Possible choice/ Answers |
| --- | --- | --- |
| **201** | Do you plan to leave the current health facility? | Yes 2. No |
|  | If Q.201 yes, when do you leave this institution? | 1. Within one year 4. 3- 4 years 2. 1-2 years 5. 4- 5 years 3. 2-3 years 6. After 5 years |
|  | Intention after leaving the institution where do you prefer to work? | 1. Working in other governmental organization 2. Work in NGO/private 3. Work in non-health institution 4. Running own business 5. Other (specify) ____________ |
|  | What kind of job are you looking for? | - 1. Job in same profession   2. Job in another profession   3. Other (specify) ___________ |

**THANK YOU FOR YOUR TIME AND COOPERATION**
